# Dietary Patterns, Alzheimer’s Disease Mortality, and Psychometric Mild Cognitive Impairment: The National Health and Nutrition Examination Survey

**DOI:** 10.1002/alz.086815

**Published:** 2025-01-09

**Authors:** Yiying Gong, Hui Chen, Jie Shen, Ting Shen, Yihong Ding, Mengxi Lu, Liyan Huang, Changzheng Yuan

**Affiliations:** ^1^ School of Public Health, the Second Affiliated Hospital, Zhejiang University School of Medicine, Hangzhou, Zhejiang China; ^2^ Harvard T.H. Chan School of Public Health, Boston, MA USA

## Abstract

**Background:**

Dietary factors play a major role in cognitive aging, but few study has compared the associations of major dietary patterns with Alzheimer’s disease (AD) mortality and psychometric mild cognitive impairment (p‐MCI).

**Method:**

We included 27,773 participants (mean age = 57.0 years, 52.9% female) from the US National Health and Nutrition Examination Survey (NHANES), 1998‐2016. We calculated five dietary pattern scores from repeated one day 24‐hour recalls, including the Healthy Eating Index (HEI‐2015), alternate Mediterranean diet score (AMED), Dietary Approach to Stop Hypertension score (DASH), the Mediterranean‐DASH Intervention for Neurodegeneration Delay diet score (MIND), and the healthful plant‐based diet index (hPDI). AD mortality was ascertained from linkage to the National Death Index through December, 2019. P‐MCI is defined as a composite cognitive function score being 1.0 standard deviation (SD) lower than the age‐, sex‐, and education‐specific norm measured in 2,713 participants in 2011‐2014. We applied Cox proportional hazard models and logistic models to assess the relations of the dietary scores to AD mortality and p‐MCI, respectively.

**Result:**

During follow‐up (median = 9.8 years), 260 participants died of AD. Higher AMED was associated with lower risk of AD mortality (HR per SD: 0.87, 0.76 to 0.99). In 2011‐2014, 432 out of 2713 participants had prevalent p‐MCI. Higher HEI‐2015 (OR per SD: 0.89, 95%CI: 0.80 to 1.00), AMED (0.84, 0.75 to 0.95), and MIND (0.81, 0.72 to 0.92) scores were associated with lower odds of p‐MCI.

**Conclusion:**

Adherence to the Mediterranean diet is associated with both lower risk of AD mortality and lower odds of p‐MCI. Our findings highlight the importance of health dietary patterns in the healthy cognitive aging.

